# Clinical outcomes of cancer-associated isolated distal deep vein thrombosis: a comparison between asymptomatic and symptomatic thrombosis—findings from the ONCO DVT Study

**DOI:** 10.1016/j.rpth.2025.102722

**Published:** 2025-03-06

**Authors:** Yoshito Ogihara, Yugo Yamashita, Takeshi Morimoto, Nao Muraoka, Michihisa Umetsu, Yuji Nishimoto, Takuma Takada, Tatsuya Nishikawa, Nobutaka Ikeda, Kazunori Otsui, Daisuke Sueta, Yukari Tsubata, Masaaki Shoji, Ayumi Shikama, Yutaka Hosoi, Yasuhiro Tanabe, Ryuki Chatani, Kengo Tsukahara, Naohiko Nakanishi, Kitae Kim, Satoshi Ikeda, Toru Sato, Takeshi Kimura, Kaoru Dohi, ONCO DVT Study Investigators

**Affiliations:** 1 Department of Cardiology and Nephrology, Mie University Graduate School of Medicine, Tsu, Japan; 2 Department of Cardiovascular Medicine, Graduate School of Medicine, Kyoto University, Kyoto, Japan; 3 Department of Clinical Epidemiology, Hyogo Medical University, Nishinomiya, Japan; 4 Division of Cardiology, Shizuoka Cancer Center, Shizuoka, Japan; 5 Division of Vascular Surgery, Department of Surgery, Tohoku University Hospital, Sendai, Japan; 6 Division of Cardiology, Osaka General Medical Center, Osaka, Japan; 7 Department of Cardiology, Tokyo Women’s Medical University, Tokyo, Japan; 8 Department of Onco-Cardiology, Osaka International Cancer Institute, Osaka, Japan; 9 Division of Cardiovascular Medicine, Toho University Ohashi Medical Center, Tokyo, Japan; 10 Department of General Internal Medicine, Kobe University Graduate School of Medicine, Kobe, Japan; 11 Department of Cardiovascular Medicine, Graduate School of Medical Sciences, Kumamoto University, Kumamoto, Japan; 12 Department of Internal Medicine, Division of Medical Oncology and Respiratory Medicine, Shimane University Faculty of Medicine, Izumo, Japan; 13 Department of Cardiovascular Medicine, National Cancer Center Hospital, Tokyo, Japan; 14 Department of Obstetrics and Gynecology, Faculty of Medicine, University of Tsukuba, Tsukuba, Japan; 15 Department of Cardiovascular Surgery, Kyorin University Faculty of Medicine, Tokyo, Japan; 16 Department of Cardiology, St. Marianna University School of Medicine, Kawasaki, Japan; 17 Department of Cardiovascular Medicine, Kurashiki Central Hospital, Kurashiki, Japan; 18 Division of Cardiology, Fujisawa City Hospital, Fujisawa, Japan; 19 Department of Cardiovascular Medicine, Graduate School of Medical Science, Kyoto Prefectural University of Medicine, Kyoto, Japan; 20 Department of Cardiovascular Medicine, Kobe City Medical Center General Hospital, Kobe, Japan; 21 Department of Cardiovascular Medicine, Nagasaki University Graduate School of Biomedical Sciences, Nagasaki, Japan; 22 Department of Cardiology, Hirakata Kohsai Hospital, Hirakata, Japan

**Keywords:** anticoagulants, edoxaban, neoplasms, recurrence, venous thrombosis

## Abstract

**Background:**

The risk of recurrent venous thromboembolism (VTE) in patients with isolated distal deep vein thrombosis (IDDVT) is generally low, particularly when IDDVT is asymptomatic. However, cancer patients with IDDVT, even asymptomatic IDDVT, may be at a higher risk of recurrent VTE.

**Objectives:**

To compare the clinical outcomes of cancer patients with asymptomatic and symptomatic IDDVT.

**Methods:**

The ONCO DVT trial is a randomized clinical trial that compared 12-month versus 3-month edoxaban treatment regimens in cancer patients with IDDVT. In this post hoc analysis, 601 patients were categorized into the asymptomatic (*n* = 479) and symptomatic (*n* = 122) groups based on IDDVT-related symptoms at diagnosis. The primary outcome was the composite of symptomatic recurrent VTE or VTE-related death at 12 months, while the major secondary outcome was major bleeding at 12 months.

**Results:**

The cumulative 12-month incidence of the primary outcome was lower in the asymptomatic group than that in the symptomatic group (2.9% vs 13.4%; *P* < .001; hazard ratio, 0.21; 95% CI, 0.10-0.47). Among the 12 patients with symptomatic recurrent VTE in the asymptomatic group, 8 (67%) had recurrent IDDVT, and 11 (92%) experienced recurrence after discontinuing anticoagulation therapy. The cumulative 12-month incidence of major bleeding was lower in the asymptomatic group than that in the symptomatic group (7.8% and 13.2%; *P* = .048).

**Conclusion:**

The risk of recurrent symptomatic VTE was lower in cancer patients with asymptomatic IDDVT than in those with symptomatic IDDVT. Most recurrent VTE events were recurrent IDDVT, with the majority occurring after discontinuing anticoagulation therapy.

## Essentials

• Cancer-associated isolated distal deep vein thrombosis (IDDVT) has unique risks and challenges.
• This study compared clinical outcomes in cancer patients with asymptomatic vs symptomatic IDDVT.
• Patients with asymptomatic IDDVT had a lower risk of recurrent symptomatic venous thromboembolism (VTE).
• Most recurrent symptomatic VTE were IDDVT and occurred after discontinuing anticoagulation.

## 1 Introduction

Previous studies reported that the risk of recurrent venous thromboembolism (VTE) was lower in patients with isolated distal deep vein thrombosis (IDDVT), involving clots below the knee vein, than in those with proximal deep venous thrombosis (DVT), particularly in asymptomatic patients [[1], [2], [3], [4]]. Current guidelines do not specifically address the management or screening of asymptomatic IDDVT, but generally emphasize a less aggressive approach to anticoagulation therapy for low-risk IDDVT [[5], [6], [7], [8]]; however, the management becomes more challenging in cancer patients. Active cancer predisposes patients to thrombotic events due to the hypercoagulable state induced by malignancy and its treatments [[9], [10], [11], [12]]. Previous studies suggested that the cumulative incidence of recurrent VTE was similar between cancer patients with IDDVT and those with proximal DVT [[13], [14], [15]] and indicated that the risk of thrombosis was higher in cancer patients with IDDVT, even those with asymptomatic IDDVT [16,17]. Nevertheless, the clinical relevance of cancer-associated asymptomatic IDDVT remains controversial, and limited data are currently available on this issue, underscoring the clinical need to establish optimal management strategies for these patients.

The ONCO DVT trial is a randomized clinical trial that assessed the efficacy and safety of 12-month versus 3-month edoxaban treatment regimens in cancer patients with IDDVT, the majority of whom were asymptomatic [18]. This post hoc analysis aimed to compare the clinical outcomes of cancer patients with asymptomatic and symptomatic IDDVT in this cohort. This analysis attempted to address clinical gaps in the limited data available and provide clinically relevant information to guide treatment strategies for cancer-associated asymptomatic IDDVT.

## 2 Methods

### 2.1 Study design

The ONCO DVT trial (NCT03895502) is an investigator-initiated, multicenter, open-label, adjudicator-blinded, superiority, randomized clinical trial that was conducted at 60 institutions in Japan. The trial compared 12-month and 3-month edoxaban treatment regimens in cancer patients with IDDVT. The study design and primary findings have been described in detail (participating investigators and trial organization are provided in Supplementary Appendix 1) [18]. Briefly, patients with active cancer and IDDVT newly diagnosed via ultrasonography were randomly assigned in a 1:1 ratio to receive the 12-month or 3-month edoxaban treatment regimens. Edoxaban was orally administered at a fixed dose of 60 mg once daily, while a lower dose (30 mg once daily) was used for patients with a creatinine clearance of 30 to 50 mL/min or a body weight of ≤60 kg or for those receiving concomitant treatment with potent P-glycoprotein inhibitors. The study was approved by the Kyoto University Institutional Review Board and the Institutional Review Boards of all participating institutions and was conducted in accordance with the principles of the Declaration of Helsinki. Details of the participating centers are provided in Supplementary Appendix 2.

### 2.2 Study population

**Figure 1:**
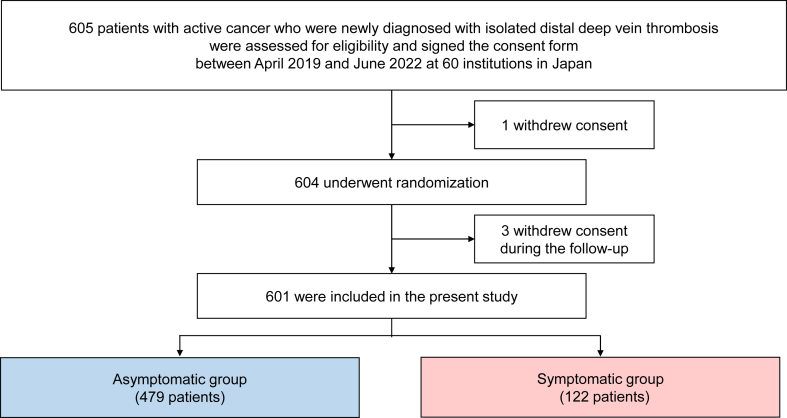
Study flow chart. Patients were divided into asymptomatic and symptomatic groups based on the presence or absence of symptoms associated with isolated distal deep vein thrombosis at diagnosis.

Between April 2019 and June 2022, 604 patients were randomized. After the exclusion of 3 patients who withdrew consent during the follow-up, 601 patients were included in the present analysis. Patients were divided into asymptomatic and symptomatic groups based on the presence or absence of IDDVT-related symptoms at diagnosis (Figure 1). A number of symptoms, such as leg swelling, local pain, warmth, change in skin color, and pain with passive leg movement (eg, Homan sign), were attributed to IDDVT by the investigator at each site [[19], [20]]. Investigators assessed symptoms through direct patient interviews, physical examinations, and reviews of medical records. The most common reason for performing ultrasonography to diagnose IDDVT in the asymptomatic group was a high-risk status with elevated D-dimer levels under chemotherapy or admission to hospital for acute diseases (47%), followed by elevated D-dimer levels before surgery (30%) (Supplementary Table S1). In Japan, D-dimer levels are used in clinical practice to identify patients at a high risk of VTE and to guide decisions to perform ultrasonography, particularly, in high-risk patients, such as those receiving chemotherapy or hospitalized for acute illness [17,21]. Additionally, patients in the asymptomatic and symptomatic groups were stratified by edoxaban treatment duration into 12-month and 3-month groups for a post hoc subgroup analysis (Supplementary Figure S1).

### 2.3 Definitions of baseline characteristics

Active cancer was defined as cancer meeting at least one of the following criteria: newly diagnosed within 6 months of randomization; receiving ongoing or recent cancer therapy (such as surgery, chemotherapy, or radiotherapy) within 6 months prior to randomization; having recurrence, local invasion, or distant metastases; or unremitted hematologic malignancy [22]. Further details on patient characteristics are provided in Supplementary Appendix 3.

### 2.4 Clinical outcomes

Clinical outcomes in the present study were assessed 12 months after enrollment and were identical to those in the primary report of the ONCO DVT trial [18]. The primary outcome was a composite of symptomatic recurrent VTE or VTE-related death at 12 months. Symptomatic recurrent VTE was defined as new or newly worsening pulmonary embolism (PE) or DVT symptoms, and new thrombi detected by imaging tests or thrombi that had worsened over time from the most recent image. VTE-related death was that diagnosed at autopsy, death following clinically severe PE, or death unexplained by any cause other than PE. The major secondary outcome was major bleeding events at 12 months. Major bleeding was defined according to the criteria of the International Society on Thrombosis and Haemostasis, which consisted of fatal bleeding, symptomatic bleeding in a critical area or organ, and bleeding causing a reduction in the level of hemoglobin by at least 2 g/dL or leading to a transfusion of at least 2 units of whole blood or red blood cells [23].

Other secondary outcomes were VTE-related death, symptomatic PE, symptomatic proximal DVT, symptomatic IDDVT, all clinically relevant bleeding events, and all-cause death at 12 months. All clinically relevant bleeding events consisted of major and nonmajor bleeding events. Clinically relevant nonmajor bleeding was defined as clinically overt bleeding (including bleeds detected only using imaging) not meeting the criteria for major bleeding, yet leading to one or more of the following: a physician-guided medical intervention, hospital admission, or further treatment for bleeding or an in-person medical examination by a physician. The cause of death was classified as resulting from VTE, bleeding, cancer, cardiovascular disease, or other causes. The persistent discontinuation of edoxaban was defined as the withdrawal of edoxaban according to the study protocol or lasting more than 14 days for any reason. An independent Clinical Events Committee, blinded to the study group assignments, adjudicated all suspected outcome events and causes of death (Supplementary Appendix 1).

### 2.5 Statistical analysis

Categorical variables are presented as numbers and percentages. Continuous variables are shown as means and standard deviations or as medians and interquartile ranges, depending on their distributions. Categorical variables were compared using the chi-squared or Fisher’s exact test. Continuous variables were compared using the Student’s *t*-test or Wilcoxon rank-sum test. The Kaplan–Meier method was used to estimate the cumulative incidence, while the log-rank test was used to assess differences in the primary and secondary outcomes between the asymptomatic and symptomatic groups. Incidence rates for clinical outcomes were calculated using the person-year method. The time period for each analysis was defined according to the respective period: (1) for the entire study period and the on-treatment period, the duration was calculated from the time of the diagnosis to the earlier of either event occurrence or the end of the respective period; and (2) for the after anticoagulation discontinuation period, the duration was calculated from the day after anticoagulation discontinuation to the earlier of either event occurrence or the end of the study period. Events occurring on the day of anticoagulation discontinuation were included in the on-treatment period but were excluded from the after anticoagulation period. Event rates are presented as the number of events per 100 patient-years, with 95% CIs calculated using Poisson exact methods. The incidence rates of primary and secondary outcomes are also presented as the number of events and percentages with 95% CI calculated using Wilson score method. A univariable Cox proportional hazards model was used to estimate the hazard ratios (HRs) and 95% CI of the asymptomatic group relative to the symptomatic group for these outcomes. A multivariable Cox proportional hazards model was not constructed due to the small number of events for the primary and secondary outcomes. As a sensitivity analysis, a subdistribution hazard model was used to estimate the HR and 95% CI of the asymptomatic group relative to the symptomatic group. In the analysis of recurrent VTE, death due to causes other than PE was treated as a competing risk. In the analysis of major bleeding, death due to causes other than bleeding was treated as a competing risk. To further investigate the effects of the treatment duration, we conducted analyses for both the entire study period and the on-treatment period. Additionally, a post hoc subgroup analysis was conducted to evaluate whether the effect of 12-month versus 3-month edoxaban treatment differed based on the presence or absence of IDDVT-related symptoms.

All statistical analyses were performed using JMP version 13.0.0 software (SAS Institute), R statistical software package version 4.4.0 (The R Foundation for Statistical Computing), and EZR (Saitama Medical Center, Jichi Medical University), a graphical user interface for R. All reported *P* values were 2-tailed, and the significance of differences was set at a *P* value of <.05.

## 3 Results

### 3.1 Patient characteristics

Among the 601 patients analyzed, 479 (80%) were in the asymptomatic group and 122 (20%) in the symptomatic group (Figure 1). Symptomatic IDDVT was more frequently detected on the left side, with swelling being the most common symptom (76%), followed by local pain (31%) (Table 1).

**Table 1:** Clinical characteristics of patients at baseline.

Variables	Asymptomatic IDDVT (*n* = 479)	Symptomatic IDDVT (*n* = 122)	*P*
Baseline characteristics			
Age (y)	71.0 ± 9.9	70.2 ± 9.8	.43
Age ≥75 y, No. (%)	200 (42)	45 (37)	.33
Men, No. (%)	131 (27)	36 (30)	.63
Body weight (kg)	55.7 ± 12.1	55.0 ± 11.2	.56
Body weight <60 kg, No. (%)	336 (70)	85 (70)	.92
Body mass index (kg/m^2^)	22.6 ± 4.2	22.4 ± 3.7	.60
Symptoms at baseline			
Swelling, No. (%)	—	93 (76)	—
Local pain, No. (%)	—	38 (31)	—
Warmth, No. (%)	—	3 (2.5)	—
Change in skin color, No. (%)	—	3 (2.5)	—
Homan sign: pain with passive leg movement, No. (%)	—	8 (6.6)	—
Site of thrombosis			
Bilateral side, No. (%)	178 (37)	45 (37)	.01
Right side, No. (%)	134 (28)	20 (16)	
Left side, No. (%)	167 (35)	57 (47)	
Cancer status			
Newly diagnosed cancer within 6 mo, No. (%)	331 (69)	58 (48)	<.001
Chemotherapy performed within 6 mo, No. (%)	200 (42)	83 (68)	<.001
Radiotherapy performed within 6 mo, No. (%)	39 (8.1)	13 (11)	.38
Recurrent cancer, No. (%)	43 (9.0)	22 (18)	.004
Metastatic disease, No. (%)	111 (23)	36 (30)	.15
ECOG performance status,^a^ No. (%)			
0	262 (55)	49 (40)	.002
1	142 (30)	39 (32)	
≥2	75 (16)	34 (28)	
Comorbidities			
Hypertension, No. (%)	210 (44)	53 (43)	.94
Diabetes, No. (%)	78 (16)	23 (19)	.50
Heart failure, No. (%)	6 (1.3)	4 (3.3)	.12
History of stroke, No. (%)	24 (5.0)	3 (2.5)	.33
History of VTE, No. (%)	21 (4.4)	12 (9.8)	.02
History of major bleeding,^b^ No. (%)	16 (3.3)	7 (5.7)	.22
Transient risk factors for VTE,^c^ No. (%)	113 (24)	38 (31)	.08
Recent surgery within 2 mo, No. (%)	66 (14)	19 (16)	.61
Laboratory tests at diagnosis			
Creatinine clearance ≤50 mL/min, No. (%)	100 (21)	31 (25)	.28
Anemia,^d^ No. (%)	305 (64)	97 (80)	<.001
Platelet count <100,000/μL, No. (%)	23 (4.8)	8 (6.6)	.43
D-dimer (μg/mL)^e^	4.9 (2.1-10.5)	6.0 (2.5-13.5)	.08
Concomitant medication			
Antiplatelet, No. (%)	39 (8.1)	9 (7.4)	.78
Steroid, No. (%)	53 (11)	24 (20)	.01
Statins, No. (%)	116 (24)	18 (15)	.03
Initial parenteral therapy with heparin	33 (6.9)	6 (4.9)	.43
Doses of edoxaban			
Standard dose of edoxaban (60 mg/d), No. (%)	124 (26)	27 (22)	.39
Lower dose of edoxaban (30 mg/d),^f^ No. (%)	355 (74)	95 (78)	
Assignment to 12-mo or 3-mo edoxaban group			
12-mo edoxaban group	243 (51)	53 (43)	.15
3-mo edoxaban group	236 (49)	69 (57)	
Duration of anticoagulation median (d)	110 (84-365)	98 (73-336)	.04

Categorical variables are presented as numbers and percentages, and continuous variables as means and standard deviations or medians and interquartile ranges based on their distributions. Categorical variables were compared with the chi-squared test when appropriate; otherwise, Fisher’s exact test was used. Continuous variables were compared using the Student’s *t*-test or Wilcoxon rank-sum test based on their distributions.

ECOG, Eastern Cooperative Oncology Group; IDDVT, isolated distal deep vein thrombosis; VTE, venous thromboembolism.

a Eastern Cooperative Oncology Group (ECOG) performance status values range from 0 to 4, with higher values indicating greater disability.

b A history of major bleeding was diagnosed if the patient had a history of International Society of Thrombosis and Haemostasis major bleeding.

c Transient risk factors for VTE included recent surgery, recent immobilization, long-distance travel, central venous catheter use, pregnancy or puerperium, recent leg trauma, fracture or burn, severe infection, and estrogen use.

d Anemia was diagnosed if the level of hemoglobin was <13 g/dL for men and <12 g/dL for women.

e Values for D-dimer were missing in 34 patients.

f Edoxaban was administered at a dose of 30 mg once daily (instead of 60 mg once daily) to patients with a creatinine clearance of 30 to 50 mL/min or a body weight of ≤60 kg or to those receiving concomitant treatment with potent P-glycoprotein inhibitors.

Cancer patients in the asymptomatic group were more often newly diagnosed within 6 months (69% vs 48%; *P* < .001), were less often under chemotherapy within 6 months (42% vs 68%; *P* < .001), less frequently had recurrence (9.0% vs 18%; *P* = .004), and had a lower Eastern Cooperative Oncology Group (ECOG) performance status (ECOG 0: 55% vs 40%; ECOG 1: 30% vs 32%; ECOG ≥2: 16% vs 28%; *P* = .002) than those in the symptomatic group. The frequency of metastatic cancer was numerically lower in the asymptomatic group (23% vs 30%; *P* = .15) (Table 1). The most common type of underlying malignancy was ovarian cancer (15%), followed by uterine (13%), lung (12%), and colorectal cancers (9.7%) (Supplementary Table S2).

### 3.2 Therapeutic management

**Figure 2:**
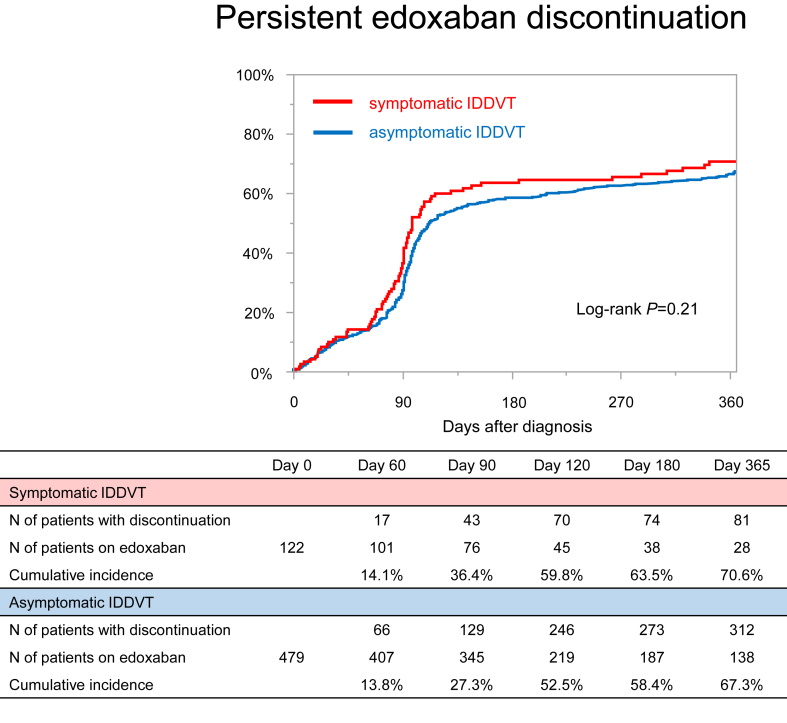
Kaplan–Meier curves for persistent edoxaban discontinuation in asymptomatic and symptomatic groups. Persistent edoxaban discontinuation was defined as the withdrawal of edoxaban according to the study protocol or lasting for more than 14 days for any reason. IDDVT, isolated distal deep vein thrombosis.

No significant differences were observed in edoxaban doses between the asymptomatic and symptomatic groups (60 mg/d: 26% vs 22%; 30 mg/d: 74% vs 78%; *P* = .39) (Table 1). There were also no significant differences in the cumulative incidence of persistent edoxaban discontinuation between the 2 groups (67.3% and 70.6% at 12 months; *P* = .21) (Figure 2). The median duration of anticoagulation therapy was significantly longer in the asymptomatic group than that in the symptomatic group (110 days [84-365] vs 98 days [73-336]; *P* = .04) (Table 1).

### 3.3 Clinical outcomes

**Figure 3:**
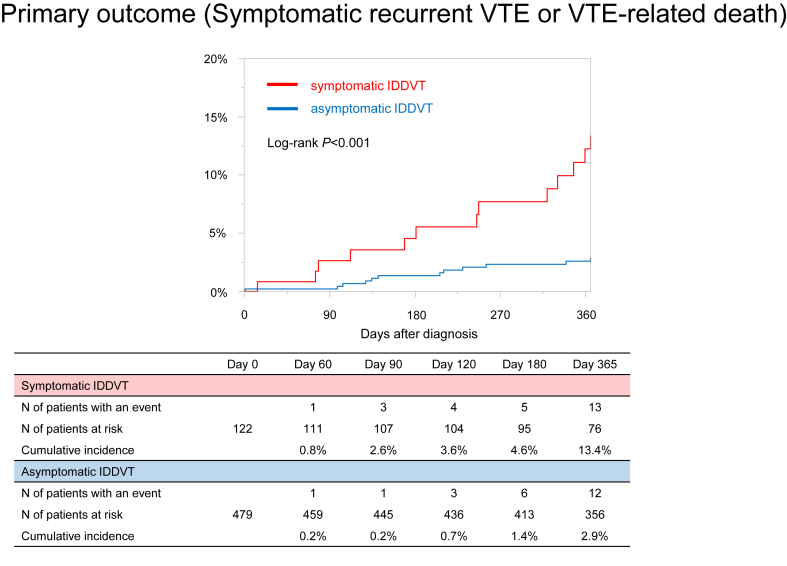
Kaplan–Meier curves for the primary outcome in asymptomatic and symptomatic groups. The primary outcome consisted of symptomatic recurrent VTE or VTE-related death. IDDVT, isolated distal deep vein thrombosis; VTE, venous thromboembolism.

The cumulative 12-month incidence of the primary outcome was lower in the asymptomatic group than that in the symptomatic group (2.9% vs 13.4%; *P* < .001; HR, 0.21; 95% CI, 0.10-0.47) (Figure 3 and Table 2). The incidence rates of the primary outcome during the entire study period were 2.89 (95% CI, 1.49-5.05) and 13.51 (95% CI, 7.19-23.10) per 100 patient-years in the asymptomatic and symptomatic groups, respectively (Table 3). The incidence rates of the primary outcome during the on-treatment period were 0.41 (95% CI, 0.01-2.31) and 5.60 (95% CI, 1.16-16.37) per 100 patient-years, whereas after the discontinuation of anticoagulation therapy, these rates increased to 6.44 (95% CI, 3.21-11.52) and 23.81 (95% CI, 11.42-43.78) per 100 patient-years (Supplementary Table S3A, B) in the asymptomatic and symptomatic groups, respectively. Among 12 patients with symptomatic recurrent VTE in the asymptomatic group, 8 (67%) developed symptomatic recurrent IDDVT (Supplementary Table S4), and 11 (92%) developed symptomatic recurrent VTE after discontinuing anticoagulation therapy. Of the 11 patients, 7 resumed edoxaban anticoagulation therapy.

**Table 2:** Cumulative incidence and hazard ratios of clinical outcomes at 12 months.

Outcomes	Asymptomatic IDDVT (*n* = 479)	Symptomatic IDDVT (*n* = 122)	HR (95% CI)
	Number of patients with an event (cumulative 12-mo incidence)	Number of patients with an event (cumulative 12-mo incidence)	
Primary outcome			
Symptomatic recurrent VTE or VTE-related death	12 (2.9)	13 (13.4)	0.21 (0.10-0.47)
Secondary outcomes			
Major bleeding	35 (7.8)	15 (13.2)	0.55 (0.31-1.03)
All clinically relevant bleeding	67 (14.8)	27 (24.0)	0.57 (0.37-0.90)
All-cause death	105 (22.2)	38 (31.4)	0.65 (0.45-0.96)

The cumulative 12-month incidence was estimated using the Kaplan–Meier method. HR and 95% CI of the asymptomatic group relative to the symptomatic group were estimated using univariable Cox proportional hazard models.

HR, hazard ratio; IDDVT, isolated distal deep vein thrombosis; VTE, venous thromboembolism.

**Table 3:** Incidence rates of clinical outcomes during the entire study period.

Outcomes	Asymptomatic IDDVT	Symptomatic IDDVT
Primary outcome		
Symptomatic recurrent VTE or VTE-related death		
Incidence rate (100 patient-years)	2.89 (1.49-5.05)	13.51 (7.19-23.10)
Time to event (d)	174 (110-249)	245 (92-339)
Secondary outcomes		
Major bleeding		
Incidence rate (100 patient-years)	8.62 (6.00-11.99)	16.18 (9.05-26.68)
Time to event (d)	82 (30-161)	28 (16-62)
All clinically relevant bleeding		
Incidence rate (100 patient-years)	17.27 (13.39-21.94)	31.80 (20.96-46.27)
Time to event (d)	57 (20-161)	25 (12-112)
All-cause death		
Incidence rate (100 patient-years)	25.04 (20.48-30.31)	38.43 (27.19-52.75)
Time to event (d)	173 (77-260)	133 (67-220)

Incidence rates were calculated using the person-year method. The time period for calculating incidence rates was defined as the duration from the time of the diagnosis to the earlier of either event occurrence or the end of the observation period. Poisson exact methods were used to calculate incidence rates with 95% CI. Continuous variables are presented as medians and interquartile ranges.

IDDVT, isolated distal deep vein thrombosis; VTE, venous thromboembolism.

**Figure 4:**
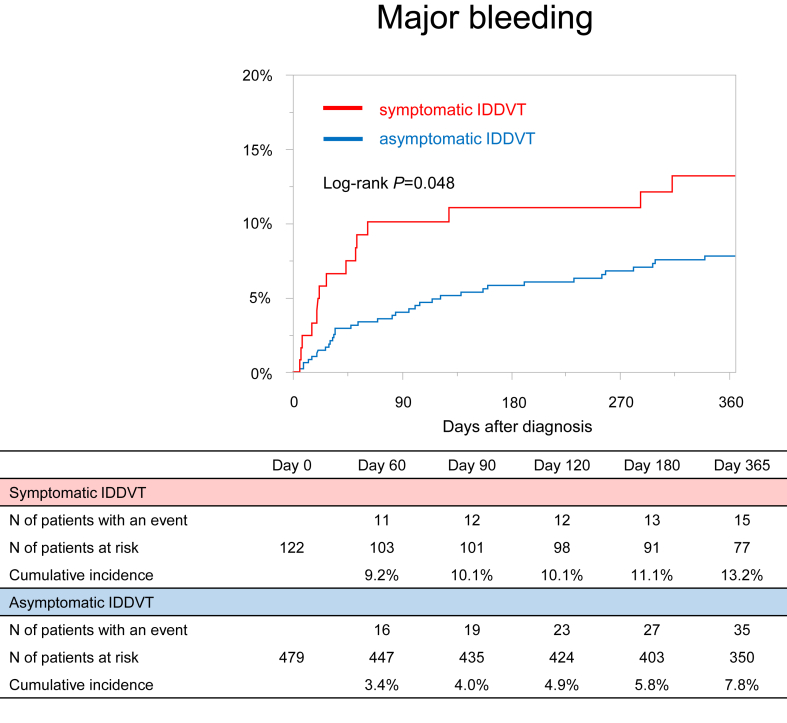
Kaplan–Meier curves for major bleeding in asymptomatic and symptomatic groups. Major bleeding was defined according to the definition of the International Society on Thrombosis and Haemostasis criteria, which consisted of fatal bleeding, symptomatic bleeding in a critical area or organ, and bleeding causing a reduction in the level of hemoglobin by at least 2 g/dL or leading to a transfusion of at least 2 units of whole blood or red cells. IDDVT, isolated distal deep vein thrombosis.

The cumulative 12-month incidence of major bleeding was lower in the asymptomatic group than in the symptomatic group (7.8% and 13.2%; *P* = .048; HR, 0.55; 95% CI, 0.31-1.03) (Figure 4 and Table 2). The incidence rates of major bleeding during the entire study period were 8.62 (95% CI, 6.00-11.99) and 16.18 (95% CI, 9.05-26.68) per 100 patient-years in the asymptomatic and symptomatic groups, respectively (Table 3).

The cumulative 12-month incidence of all-cause death was also lower in the asymptomatic group than that in the symptomatic group (22.2% and 31.4%; *P* = .02; HR, 0.65; 95% CI, 0.45-0.96) (Supplementary Figure S2 and Table 2). The incidence rates of all-cause death during the entire study period were 25.04 (95% CI, 20.48-30.31) and 38.43 (95% CI, 27.19-52.75) per 100 patient-years in the asymptomatic and symptomatic groups, respectively (Table 3).

The results of a sensitivity analysis using a subdistribution hazard model were consistent with those described above. The asymptomatic group showed a significantly lower risk of the primary outcome (the entire study period: HR, 0.23; 95% CI, 0.10-0.50; the on-treatment period: HR, 0.08; 95% CI, 0.01-0.79) and a numerically lower risk of major bleeding (the entire study period: HR, 0.57; 95% CI, 0.31-1.04; the on-treatment period: HR, 0.55; 95% CI, 0.28-1.08).

As for the post hoc subgroup analysis, in the asymptomatic subgroup, no primary outcome events occurred in the 12-month edoxaban group, while 12 of the 236 patients in the 3-monath edoxaban group experienced the primary outcome (0% vs 5.1%). In the symptomatic subgroup, the primary outcome occurred in 3 of the 53 patients in the 12-month edoxaban group and in 10 of the 69 patients in the 3-monath edoxaban group (5.7% vs 14.5%; odds ratio [OR], 0.35; 95% CI, 0.09-1.36) (Supplementary Figure S3). Although an interaction analysis could not be conducted due to the absence of events in the 12-month edoxaban group of asymptomatic patients, these results showed a similar trend to the overall findings in the primary study. For major bleeding, an interaction analysis was feasible. In the asymptomatic subgroup, major bleeding occurred in 20 of the 243 patients in the 12-month edoxaban group and in 15 of the 236 patients in the 3-monath edoxaban group (8.2% vs 6.4%; OR, 1.32; 95% CI, 0.66-2.65). In the symptomatic subgroup, major bleeding occurred in 8 of the 53 patients in the 12-month edoxaban group and in 7 of the 69 patients in the 3-monath edoxaban group (15.1% vs 10.1%; OR, 1.57; 95% CI, 0.53-4.66). No significant interaction was found between the subgroups in terms of the effect of 12-month edoxaban treatment relative to 3-month edoxaban treatment on major bleeding (*P*_interaction_ = .79) (Supplementary Figure S3).

## 4 Discussion

The main results of the present study are as follows: (1) the risk of recurrent symptomatic VTE was lower in cancer patients with asymptomatic IDDVT than in those with symptomatic IDDVT; (2) in the asymptomatic group, most recurrent VTE events were IDDVT, with the majority occurring after the discontinuation of anticoagulation therapy.

There have been no studies on cancer patients with asymptomatic IDDVT that revealed the incidence of recurrent VTE and directly compared the clinical outcomes of asymptomatic and symptomatic IDDVT. Observational studies on and a meta-analysis of cancer patients with symptomatic IDDVT reported that the recurrence rates of VTE ranged between 5.65 and 13.21 per 100 patient-years [[24], [25], [26]], which is consistent with the present results of 13.51 per 100 patient-years despite differences in anticoagulation regimens. In contrast, the recurrence rate in our asymptomatic group was significantly lower at 2.89 per 100 patient-years. These results suggest that the risk of recurrent VTE was lower in cancer patients with asymptomatic IDDVT, which may be attributed to the presence of a thrombus in the subacute phase and low-grade inflammation associated with the thrombus formation. Furthermore, the present study showed that among cancer patients with asymptomatic IDDVT, there were fewer cases of recurrent and metastatic cancer, indicating a lower prevalence of advanced-stage cancer. Previous studies reported that the risk of VTE recurrence was higher in patients with advanced-stage cancers [27,28], which supports the lower risk of VTE recurrence observed in patients with asymptomatic IDDVT in the present study. On the other hand, the results obtained herein also showed that most recurrent thrombotic events in asymptomatic IDDVT were IDDVT, which may limit the benefits of anticoagulant therapy in these patients. However, it is important to note that most of these events occurred after the discontinuation of edoxaban, accounting for 92% of cases, with a recurrence rate of 6.44 per 100 patient-years. The worsening of DVT in the treatment course of cancer, whether as a new symptomatic event in another vein or as an extension of previous IDDVT, may lead to the resumption of anticoagulation. This may, in turn, result in the temporal interruption or delay of cancer treatment, including chemotherapy and scheduled surgery, potentially affecting the overall prognosis of patients [9]. Therefore, the careful consideration of the continuation or discontinuation of anticoagulation may be warranted to avoid complications that may have a negative impact on cancer treatment.

Previous studies on cancer patients with symptomatic IDDVT reported incidence rates of between 1.97 and 4.08 per 100 patient-years and between 30.22 and 38.79 per 100 patient-years for major bleeding and all-cause death, respectively [[24], [25], [26]]. In the present study, the symptomatic group had a higher rate of major bleeding compared with the previously reported study, whereas the all-cause mortality in the present study was comparable with that in the previously reported study (16.18 per 100 patient-years and 38.43 per 100 patient-years, respectively). In contrast, the asymptomatic group had lower rates of bleeding events and all-cause death (8.62 per 100 patient-years and 25.04 per 100 patient-years, respectively), which may reflect less-advanced cancer stages and the better overall health status of patients analyzed in the present study. Therefore, while the benefits of continued anticoagulation in patients with asymptomatic IDDVT appear to be less pronounced due to their lower risk of thrombotic events, there may be fewer concerns regarding the risk of bleeding with prolonged anticoagulation therapy. Based on these findings, the decision to continue or discontinue anticoagulation therapy in cancer patients with asymptomatic IDDVT needs to be carefully considered according to the balance between potential benefits and risks in individual patients.

The present study had several limitations. This analysis used data from a multicenter study in Japan. Therefore, demographics and clinical outcomes may differ from those outside Japan, potentially limiting the generalizability of the results obtained. Furthermore, the use of D-dimer levels to identify high-risk patients for ultrasonography may have introduced a bias because D-dimer lacks specificity in cancer patients. Elevated D-dimer levels, often affected by nonthrombotic factors, may have led to an overdiagnosis in the asymptomatic group. However, ultrasonography was typically performed for high-risk patients rather than as part of routine screening. This may have minimized the risk of an overdiagnosis and ensured the inclusion of clinically significant cases. Moreover, the adjudication process for IDDVT-related symptoms was conducted by investigators at each site based on direct patient interviews, physical examinations, and reviews of medical records. While this approach ensured the systematic classification of symptomatic and asymptomatic patients, there remains a potential for subjectivity, particularly in cases with mild or nonspecific symptoms. This limitation may have led to the underrepresentation of symptomatic cases and needs to be considered when interpreting results. Another limitation is that, although a sensitivity analysis using a subdistribution hazard model revealed consistent results for symptomatic recurrent VTE, the small number of primary outcome events limited the ability to perform a multivariable model in order to adjust for baseline differences, such as edoxaban dosing and cancer burden, between the asymptomatic and symptomatic groups. These unadjusted differences may have affected the incidence rates for recurrent VTE and bleeding. Despite these limitations, the present results suggest that an asymptomatic status still provides meaningful insights into the clinical outcomes of this population.

## 5 Conclusions

The risk of recurrent symptomatic VTE was lower in cancer patients with asymptomatic IDDVT than in those with symptomatic IDDVT. Most recurrent VTE events were IDDVT and occurred after the discontinuation of anticoagulation therapy. These results need to be interpreted with caution due to the potential impact of confounding factors, particularly cancer burden.
