# Supplementary material for: Clinical outcomes of cancer-associated isolated distal deep vein thrombosis: a comparison between asymptomatic and symptomatic thrombosis—findings from the ONCO DVT Study
Source: Res Pract Thromb Haemost. 2025 Mar 6;9(2):102722. doi: 10.1016/j.rpth.2025.102722 (PMC11992421; doi:10.1016/j.rpth.2025.102722)
Supplement: Supplementary Appendix [file mmc1.docx]

**Supplementary Materials**

# **Supplementary Appendixes**

**Supplementary Appendix 1: Participating Investigators and Trial Organization**

**Principal Investigator:** Yugo YAMASHITA, Department of Cardiovascular Medicine, Graduate School of Medicine and Faculty of Medicine Kyoto University

**Protocol Committee:** Takeshi KIMURA (Co-Principal Investigator), Department of Cardiovascular Medicine, Graduate School of Medicine and Faculty of Medicine Kyoto University; Makoto MO, Department of Cardiovascular Surgery, Yokohama Minami Kyousai Hospital; Hisashi GOTO, Department of General Surgery/Department of Cardiovascular Medicine, Tohoku University; Daisuke SUETA, Department of Cardiovascular Medicine, Kumamoto University; Yugo YAMASHITA, Department of Cardiovascular Medicine, Graduate School of Medicine and Faculty of Medicine Kyoto University

**Steering Committee:** Norikazu YAMADA, Department of Cardiovascular Medicine, Kuwana City Medical Center; Taro SHIGA, Department of Cardiovascular Medicine, Cancer Institute Hospital; Tsuyoshi YAMAMOTO, Department of Cardiovascular Intensive Care, Nippon Medical School Hospital; Masafumi FUJITA, Department of Onco-Cardiology, Osaka International Cancer Institute; Kazunori OTSUI, Department of General Internal Medicine, Kobe University Hospital; Kenichi TSUJITA, Department of Cardiovascular Medicine, Kumamoto University; Satoshi IKEDA, Department of Cardiovascular Medicine, Nagasaki University; Norimichi KOITABASHI, Department of Cardiovascular Medicine, Gunma University; Shinji HISATAKE, Department of Cardiovascular Medicine, Toho University; Naohiko NAKANISHI, Department of Cardiovascular Medicine, Kyoto Prefectural University of Medicine; Kentaro JUJO, Department of Cardiovascular Medicine, Tokyo Women’s Medical University; Koichiro SUGIMURA, Department of General Surgery/Department of Cardiovascular Medicine, Tohoku University; Ryoji TAKEDA, Department of Vascular Surgery, Rakuwakai Otowa Hospital; Reo HATA, Department of Cardiovascular Medicine, Kurashiki Central Hospital; Kazushige KADOTA, Department of Cardiovascular Medicine, Kurashiki Central Hospital; Toru TAKASE, Department of Cardiovascular Medicine, Kindai University Hospital; Shunichi MIYAZAKI, Department of Cardiovascular Medicine, Kindai University Hospital; Seiichi HIRAMORI, Department of Cardiovascular Medicine, Kokura Memorial Hospital; Kenji ANDO, Department of Cardiovascular Medicine, Kokura Memorial Hospital; Kite KIM, Department of Cardiovascular Medicine, Kobe City Medical Center General Hospital; Yutaka FURUKAWA, Department of Cardiovascular Medicine, Kobe City Medical Center General Hospital; Jiro SAKAMOTO, Department of Cardiovascular Medicine, Tenri Hospital; Masaharu AKAO, Department of Cardiovascular Medicine, NHO Kyoto Medical Center

**Research Operations Staff:** Yusuke YOSHIKAWA, Department of Cardiovascular Medicine, Graduate School of Medicine and Faculty of Medicine Kyoto University

**Clinical Events Committee:** Yasuhiro HAMATANI, Department of Cardiovascular Medicine, NHO Kyoto Medical Center; Kensuke TAKABAYASHI, Department of Cardiovascular Medicine, Hirakata Kohsai Hospital; Yuji NISHIMOTO, Department of Cardiovascular Medicine, Hyogo Prefectural Amagasaki General Medical Center; Yukiko NAKANO, Department of Cardiovascular Medicine, Graduate School of Medicine and Faculty of Medicine Kyoto University

**Data Safety Monitoring Committee:** Mitsuru ABE Department of Cardiovascular Medicine, NHO Kyoto Medical Center; Hidenori YAKU, Department of Cardiovascular Medicine, Mitsubishi Kyoto Hospital

**Clinical Research Organization:** MID, Inc. and Department of Cardiovascular Medicine, Graduate School of Medicine and Faculty of Medicine Kyoto University

**Monitoring Officers:** Yasuaki TAKEJI, Department of Cardiovascular Medicine, Kyoto University Hospital; Yusuke YOSHIKAWA, Department of Cardiovascular Medicine, Kyoto University Hospital

**Auditors:** Chikashi TAKEDA, Department of Pharmacoepidemiology, Kyoto University Graduate School of Medicine and Public Health; Aki KUWAUCHI, Department of Pharmacoepidemiology, Kyoto University Graduate School of Medicine and Public Health

**Principal Statistician:** Takeshi MORIMOTO, Department of Clinical Epidemiology, Hyogo College of Medicine

**Supplementary Appendix 2: Participating Centers**

Department of Cardiovascular Medicine, Kyoto University Hospital (Yugo YAMASHITA), Department of Onco-Cardiology, Osaka International Cancer Institute (Masafumi FUJITA), Department of Cardiovascular Medicine, Saiseikai Noe Hospital (Ichiro KOUCHI), Department of Cardiology, Osaka Red Cross Hospital (Tsukasa INADA), Department of Cardiovascular Medicine, Japanese Red Cross Otsu Hospital (Kazuaki KAITANI), Department of Cardiovascular Medicine, Kakogawa Central City Clinics (Hiroaki NAKAMURA), Department of Cardiovascular Medicine, Cancer Institute Hospital (Taro SHIGA), Department of Vascular Surgery, Kansai Medical University Medical Center (Nobuko YAMAMOTO), Department of Cardiovascular Medicine, University Hospital Kyoto Prefectural University of Medicine (Satoaki MATOBA), Department of Cardiovascular Surgery, Kyorin University Faculty of Medicine (Yutaka HOSOI), Department of Cardiovascular Medicine, Kindai University Hospital (Gaku NAKAZAWA), Department of Cardiovascular Medicine, Kumamoto University Hospital (Daisuke SUETA), Department of Cardiovascular Medicine, Kurashiki Central Hospital (Kazushige KADOTA), Department of Cardiovascular Surgery, Kurume University Hospital (Shinichi HIROMATSU), Department of Cardiovascular Medicine, Kuwana City Medical Center (Norikazu YAMADA), Department of Cardiovascular Medicine, Gunma University (Norimichi KOITABASHI), Department of Cardiovascular Medicine, Kobe City Medical Center General Hospital (Yutaka FURUKAWA), Department of General Internal Medicine, Kobe University Hospital (Kazunori OTSUI), Department of Cardiovascular Medicine, Kohka Public Hospital (Tomohiro DOUKA), Department of Cardiovascular Surgery, Fukushima Medical University Hospital (Daiki WAKAMATSU), Department of Cardiovascular Medicine, Kokura Memorial Hospital (Kenji ANDO), Department of General Internal Medicine / Department of Cardiovascular Medicine, National Cancer Center Hospital (Masaaki SHOJI), Department of Cardiovascular Medicine, NHO Okayama Medical Center (Hiroto SHIMOKAWAHARA), Department of Cardiovascular Medicine, NHO Kyoto Medical Center (Kosuke DOI), Department of Cardiovascular Medicine, Saiseikai Yokohamashi Nanbu Hospital (Tsutomu ENDO), Department of Cardiovascular Surgery, Saiseikai Wakayama Hospital (Atsutoshi HATADA), Department of Cardiovascular Medicine, Saku Central Hospital Advanced Care Center (Yoshikazu YAZAKI), Department of Cardiovascular Medicine, Shiga General Hospital (Takeshi UENO), Department of Cardiovascular Medicine, Shizuoka Cancer Center (Nao MURAOKA), Department of Cardiovascular Medicine, Shizuoka City Shizuoka Hospital (Ryuzo NAWATA), Department of Respiratory Medicine and Clinical Oncology, Shimane University Hospital (Yukari TSUBATA), Department of Cardiovascular Medicine, Shimada General Medical Center (Yoshiaki TSUYUKI), Department of Cardiology, St. Marianna University School of Medicine (Yasuhiro TANABE), Department of Cardiovascular Medicine, Medical Research Institute Kitano Hospital (Moriaki INOKO), Department of Obstetrics and Gynecology, University of Tsukuba Hospital (Toyomi SATO), Department of Cardiovascular Medicine, Tenri Hospital (Toshihiro TAMURA), Department of Cardiovascular Medicine, Tokyo Women’s Medical University Hospital (Yuichiro MINAMI), Department of Cardiovascular Medicine, Tokyo Metropolitan Tama Medical Center (Hiroyuki TANAKA), Department of Cardiovascular Medicine, Toho University Ohashi Medical Center (Nobutaka IKEDA), Department of Cardiovascular Medicine, Toho University Omori Medical Center (Shinji HISATAKE), Department of General Surgery, Tohoku University Hospital (Hisashi GOTO), Department of Cardiovascular Medicine, Nagasaki University Hospital (Koji MAEMURA), Department of Obstetrics and Gynecology, Nara Medical University Hospital (Ryuji KAWAGUCHI), Department of Cardiovascular Intensive Care, Nippon Medical School Hospital (Tsuyoshi YAMAMOTO), Department of Cardiovascular Medicine, Japanese Red Cross Wakayama Medical Center (Shojiro TATSUSHIMA), Department of Cardiovascular Medicine, Hyogo Prefectural Amagasaki General Medical Center (Yukihiro SATO), Department of Cardiovascular Medicine, Hirakata Kohsai Hospital (Shoji KITAGUCHI), Department of Cardiovascular Medicine, Fukui Prefectural Hospital (Susumu FUJINO), Department of Vascular Surgery, Saiseikai Yahata General Hospital (Shinsuke MII), Department of Cardiovascular Medicine, Fujisawa City Hospital (Kengo TSUKAHARA), Department of Cardiovascular Medicine, Makiminato Central Hospital (Naoya MAEHIRA), Department of Cardiovascular Medicine, Mie University Hospital (Kaoru DOHI), Department of Cardiovascular Medicine, Mitsubishi Kyoto Hospital (Takafumi YOKOMATSU), Department of Cardiovascular Medicine, Japanese Red Cross Musashino Hospital (Takashi ASHIKAGA), Department of Cardiovascular Surgery, Yokohama Minami Kyousai Hospital (Makoto MO), Hospital Department of Cardiovascular Medicine, Yokohama Rosai Hospital (Kazuhiko YUMOTO), Department of Vascular Surgery, Rakuwakai Otowa Hospital (Ryoji TAKEDA), Department of Cardiovascular Medicine, Niigata University Graduate School of Medicine and Dentistry (Shinya FUJIKI), Department of Internal Medicine, Niigata Cancer Center Niigata Hospital (Yuji OKURA), Department of Surgery of the Lower Gastrointestinal Surgery, Hyogo College of Medicine (Jihyung SONG)

**Supplementary Appendix 3: Definition of baseline characteristics**

Eastern Cooperative Oncology Group (ECOG) performance status (PS): 0, Fully active, at pre-disease performance levels without restriction. 1. Restricted physically strenuous activity, but ambulatory and able to carry out work of a light and sedentary nature. 2. Ambulatory and capable of all self-care, but unable to carry out any work activities. Up and about more than 50% of waking hours. 3. Capable of only limited self-care, confined to bed or a chair more than 50% of waking hours. 4. Completely disabled. Cannot carry out any self-care. Totally confined to bed or a chair. Diabetes: Blood glucose level ≥200 mg/dl 2 or more hours after loading in a glucose tolerance test, casual blood glucose ≥200 mg/dl, fasting blood glucose ≥126 mg/dl, or Hemoglobin A1c ≥6.5%. Even when the above tests are not performed, diabetes is defined if the patient has already been clinically diagnosed with diabetes or is taking medication to treat diabetes. Heart failure: Cases that satisfy any of the following criteria are defined as heart failure: a history of hospitalization for heart failure, clinical heart failure symptoms at New York Heart Association II (can walk on flat ground but cannot jog) or higher, left ventricular ejection fraction <40%. History of major bleeding: Cases that satisfy any of the following criteria are defined as having a history of major bleeding. A history of bleeding into vital organs, a history of bleeding that required a blood transfusion, a history of bleeding with a reduction in the hemoglobin level of ≥2 g/dl, or a history of bleeding that required fluid transfusion, vasopressors, or surgical treatment. Transient risk factors for venous thromboembolism: defined as including recent surgery, recent immobilization, long-distance travel, central venous catheter use, pregnancy or puerperium, recent leg trauma, fracture or burn, severe infection, and estrogen use. Anemia: defined as a hemoglobin level of <13 g/dL for men and <12 g/dL for women. Creatinine clearance (Ccr): Ccr levels were calculated using the Cockroft–Gault formula, as follows: Ccr (mL/min) = [(140 - age) × (body weight in kg)] / (72 × creatinine) × (0.85 if female)]. The Ccr cut-off of 50 mL/min was predefined based on previous studies [1-3].

**Supplementary Tables**

**Supplementary Table S1: Reasons for conducting ultrasonography at baseline***

| **Reasons for conducting ultrasonography, No. (%)** | **Total**  **(N=601)** | **Asymptomatic IDDVT**  **(N=479)** | **Symptomatic**  **IDDVT**  **(N=122)** |
| --- | --- | --- | --- |
| Suspected DVT based on symptoms | 122 (20) | 0 (0) | 122 (100) |
| High risk status with elevated D-dimer levels** | 227 (38) | 227 (47) | 0 (0) |
| Elevated D-dimer levels before surgery | 144 (24) | 144 (30) | 0 (0) |
| Preoperative screening | 43 (7.2) | 43 (9.0) | 0 (0) |
| Elevated D-dimer levels after surgery | 42 (7.0) | 42 (8.8) | 0 (0) |
| Non-preoperative screening | 11 (1.8) | 11 (2.3) | 0 (0) |
| Incidental detection by imaging examinations other than ultrasonography | 5 (0.8) | 5 (1.0) | 0 (0) |
| Others | 7 (1.2) | 7 (1.5) | 0 (0) |

* This table was created by referring to the supplementary material of the primary report [4].

**High risk status included on-going chemotherapy and admission to hospital for acute diseases.

Categorical variables are presented as numbers and percentages.

DVT, deep vein thrombosis; IDDVT, isolated distal deep vein thrombosis.

**Supplementary Table S2. Types of cancer at baseline.**

| **Types of cancer, No. (%)** | **Total**  **(N=601)** | **Asymptomatic IDDVT**  **(N=479)** | **Symptomatic IDDVT**  **(N=122)** |
| --- | --- | --- | --- |
| Ovary | 90 (15) | 76 (16) | 14 (11) |
| Uterus | 81 (13) | 65 (14) | 16 (13) |
| Lung | 72 (12) | 58 (12) | 14 (11) |
| Colon | 58 (9.7) | 49 (10) | 9 (7.4) |
| Pancreas | 53 (8.8) | 37 (7.7) | 16 (13) |
| Stomach | 33 (5.5) | 24 (5.0) | 9 (7.4) |
| Blood | 33 (5.5) | 23 (4.8) | 10 (8.2) |
| Breast | 33 (5.5) | 25 (5.2) | 8 (6.6) |
| Bladder | 23 (3.8) | 18 (3.8) | 5 (4.1) |
| Kidney/ureter | 13 (2.2) | 9 (1.9) | 4 (3.3) |
| Prostate | 12 (2.0) | 9 (1.9) | 3 (2.5) |
| Brain | 11 (1.8) | 7 (1.5) | 4 (3.3) |
| Esophagus | 11 (1.8) | 11 (2.3) | 0 (0) |
| Skin | 10 (1.7) | 10 (2.1) | 0 (0) |
| Liver | 10 (1.7) | 7 (1.5) | 3 (2.5) |
| Gall bladder/bile duct | 6 (1.0) | 5 (1.0) | 1 (0.8) |
| Thyroid gland | 1 (0.2) | 1 (0.2) | 0 (0) |
| Multiple | 12 (2.0) | 11 (2.3) | 1 (0.8) |
| Others | 39 (6.5) | 34 (7.1) | 5 (4.1) |

Categorical variables are presented as numbers and percentages.

IDDVT, isolated distal deep vein thrombosis.

**Supplementary Table S3A. Incidence rates of clinical outcomes for the on-treatment period.**

| **Outcomes** | **Asymptomatic IDDVT** | **Symptomatic IDDVT** |
| --- | --- | --- |
| **Primary outcome** | | |
| Symptomatic recurrent VTE or VTE-related death | | |
| Incidence rate,　100 patient-years | 0.41 (0.01–2.31) | 5.60 (1.16–16.37) |
| Time to event, days | - | - |
| **Secondary outcomes** | | |
| Major bleeding | | |
| Incidence rate,　100 patient-years | 11.75 (7.81–16.99) | 23.00 (11.88–40.17) |
| Time to event, days | 51 (23–120) | 22 (10–51) |
| All clinically relevant bleeding | | |
| Incidence rate,　100 patient-years | 23.85 (17.97–31.05) | 46.94 (30.08–69.85) |
| Time to event, days | 40 (15–122) | 22 (10–60) |
| All-cause death | | |
| Incidence rate,　100 patient-years | 5.80 (3.17–9.72) | 9.34 (3.03–21.79) |
| Time to event, days | 118 (53–236) | 105 (79–194) |

Incidence rates were calculated using the person-year method during anticoagulation therapy. The time period for calculating incidence rates was defined as the duration from the time of the diagnosis to the earlier of either event occurrence or anticoagulation discontinuation. Events occurring on the day of discontinuation were included. Poisson exact methods were used to calculate incidence rates with 95%CI. Continuous variables are presented as medians and interquartile ranges.

The median time to an event for the primary outcomes was not calculated due to the limited number of events.

CI, confidence interval; IDDVT, isolated distal deep vein thrombosis; VTE, venous thromboembolism.

**Supplementary Table S3B. Incidence rates of clinical outcomes after anticoagulation therapy discontinuation.**

| **Outcomes** | **Asymptomatic IDDVT** | **Symptomatic IDDVT** |
| --- | --- | --- |
| **Primary outcome** | | |
| Symptomatic recurrent VTE or VTE-related death | | |
| Incidence rate,　100 patient-years | 6.44 (3.21–11.52) | 23.81 (11.42–43.78) |
| Time to event, days | 105 (42–164) | 163 (72–273) |
| **Secondary outcomes** | | |
| Major bleeding | | |
| Incidence rate,　100 patient-years | 4.24 (1.70–8.73) | 7.52 (1.55–21.98) |
| Time to event, days | 29 (1–180) | 66 (8–244) |
| All clinically relevant bleeding | | |
| Incidence rate,　100 patient-years | 7.76 (4.01–13.55) | 9.06 (1.87–26.48) |
| Time to event, days | 120 (5–186) | 120 (66–220) |
| All-cause death | | |
| Incidence rate,　100 patient-years | 46.88 (37.28–58.19) | 60.44 (39.83–87.93) |
| Time to event, days | 61 (17–151) | 43 (15–135) |

Incidence rates were calculated using the person-year method. The time period for calculating incidence rates was defined as the duration from the day after anticoagulation therapy discontinuation to the earlier of either event occurrence or the end of the observation period. Events occurring on the day of discontinuation were excluded. Poisson exact methods were used to calculate incidence rates with 95%CI. Continuous variables are presented as medians and interquartile ranges.

CI, confidence interval; IDDVT, isolated distal deep vein thrombosis; VTE, venous thromboembolism.

**Supplementary Table S4: Clinical outcomes at 12 months**

| **Outcomes** | **Asymptomatic IDDVT**  **(N=479)** | **Symptomatic IDDVT**  **(N=122)** |
| --- | --- | --- |
| **Primary outcome** |  |  |
| Symptomatic recurrent VTE or VTE-related death | 12 (2.5% [1.4–4.3%]) | 13 (10.7% [6.3–17.4%]) |
| **Secondary outcomes** |  |  |
| VTE-related deaths | 0 (0% [0–0.8%]) | 0 (0% [0–3.1%]) |
| Symptomatic PE with or without DVT | 1 (0.2% [0.0–1.2%]) | 2 (1.6% [0.5–5.8%]) |
| Symptomatic DVT only | 11 (2.3% [1.3–4.1%]) | 11 (9.0% [5.1–15.4%]) |
| Symptomatic proximal DVT | 2 (0.4% [0.1–1.5%]) | 5 (4.1% [1.8–9.2%]) |
| Symptomatic IDDVT | 8 (1.7% [0.8–3.3%]) | 6 (4.9% [2.2–10.3%]) |
| Others | 1 (0.2% [0.0–1.2%]) | 0 (0% [0–3.1%]) |
| Major bleeding | 35 (7.3% [5.3–10.0%]) | 15 (12.3% [7.6–19.3%]) |
| All clinically relevant bleeding | 67 (14.0% [11.2–17.4%]) | 27 (22.1% [15.7–30.3%]) |
| All-cause death | 105 (21.9% [18.4–25.8%]) | 38 (31.1% [23.6–39.8%]) |

Clinical outcomes are presented as numbers of events and incidences with 95%CI, which were calculated using Wilson's score method.

DVT, deep venous thromboembolism; IDDVT, isolated distal deep vein thrombosis; PE, pulmonary embolism; VTE, venous thromboembolism.

**Supplementary Figure**


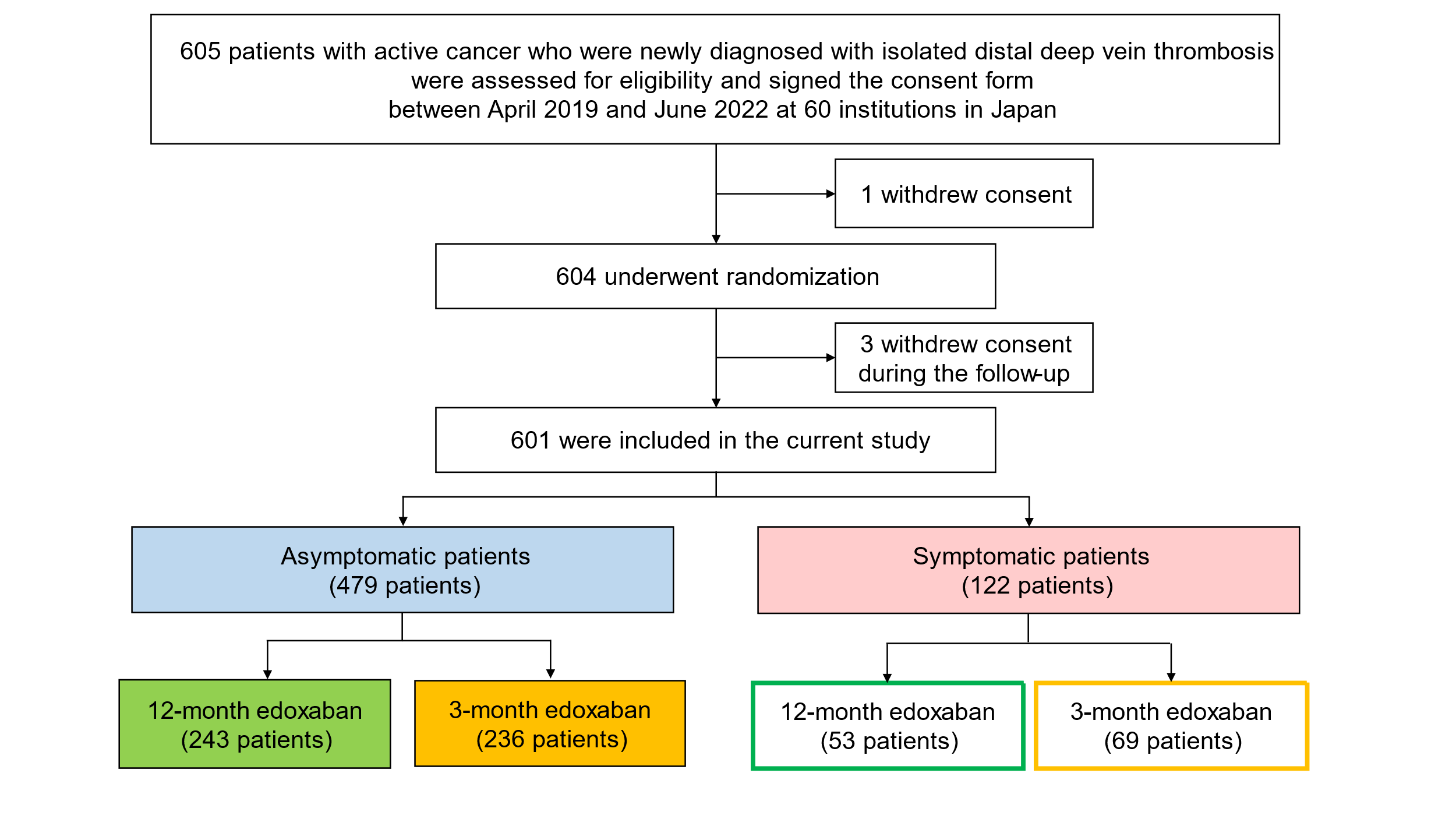
**Supplementary Figure S1: Flow chart of subgroup analysis.**

Patients were divided into asymptomatic and symptomatic groups based on the presence or absence of symptoms associated with isolated distal deep vein thrombosis at diagnosis. Additionally, patients in the asymptomatic and symptomatic groups were stratified by edoxaban treatment duration into 12-month and 3-month groups for a post-hoc subgroup analysis.

**Supplementary Figure S2: Kaplan–Meier curves for all-cause death in asymptomatic and symptomatic groups.**


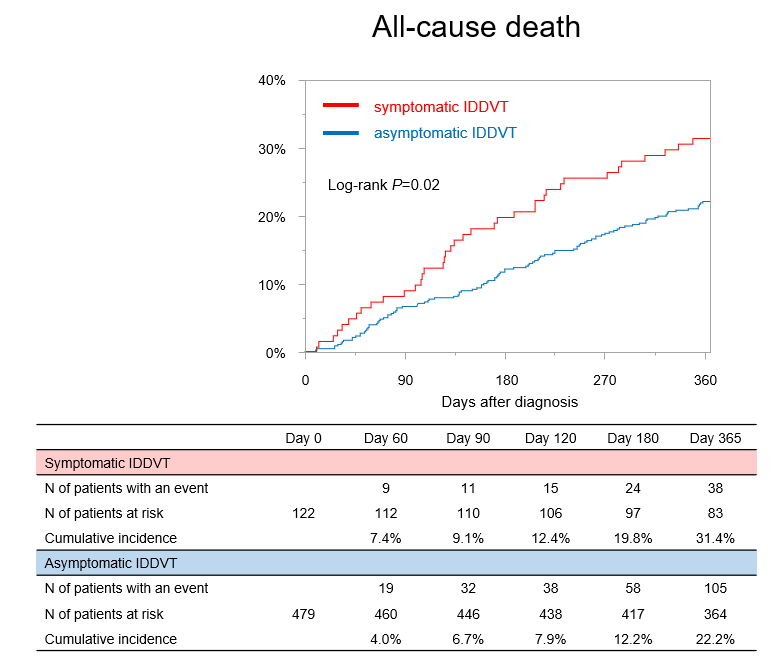


IDDVT, Isolated distal deep vein thrombosis.

**Supplementary Figure S3: Forest plots for each endpoint.**


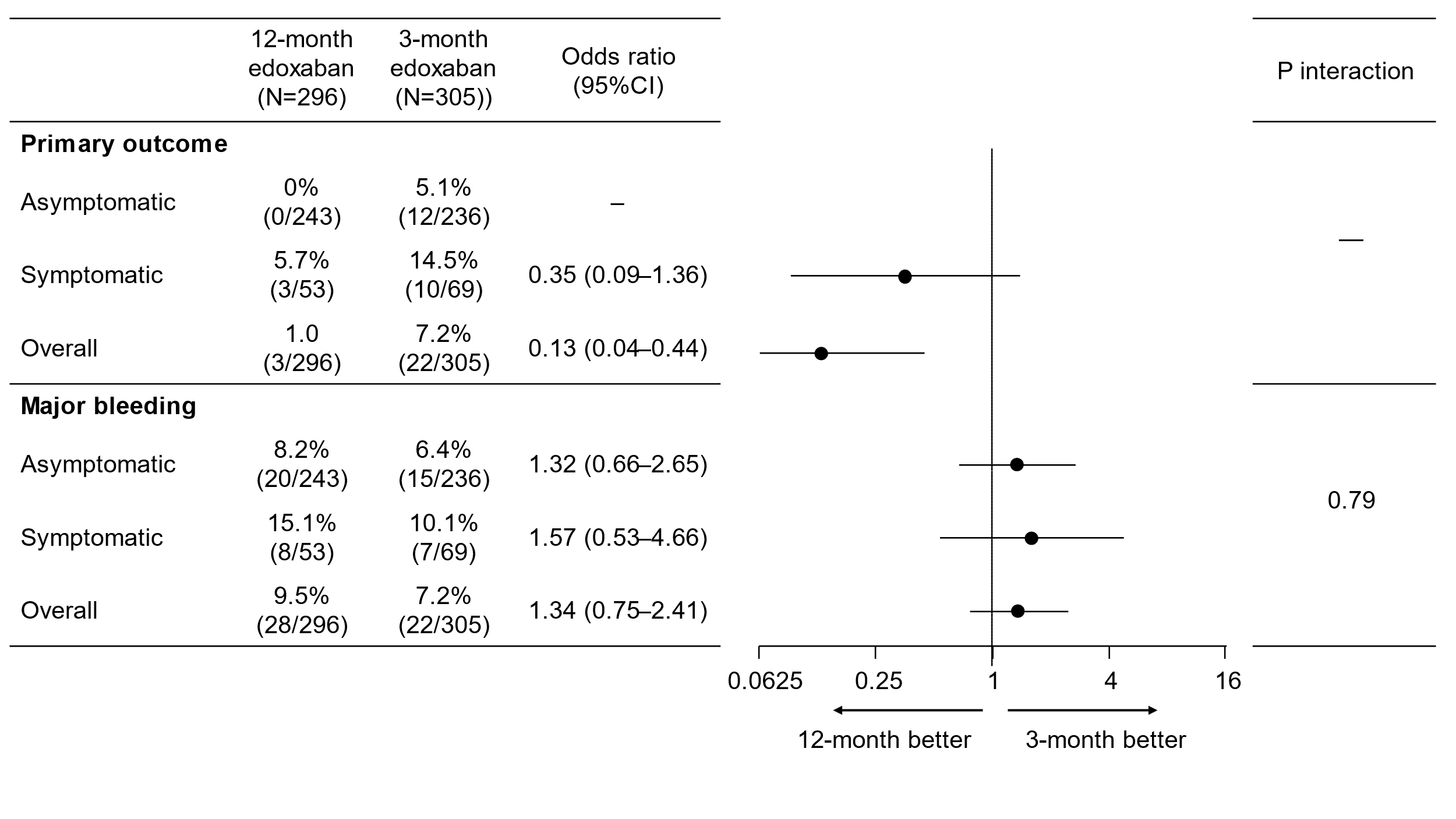


The odds ratios for each outcome in the 12-month and 3-month edoxaban groups are presented for the asymptomatic and symptomatic subgroups. The primary outcome was defined as a composite of symptomatic recurrent VTE or VTE-related death at 12 months.

VTE, venous thromboembolism.

**Supplementary References**

[1] Bohula EA, Giugliano RP, Ruff CT, Kuder JF, Murphy SA, Antman EM, et al. Impact of Renal Function on Outcomes With Edoxaban in the ENGAGE AF-TIMI 48 Trial. Circulation. 2016; 134: 24-36. https://doi.org/10.1161/circulationaha.116.022361.

[2] Yoshida T, Nakamura A, Funada J, Amino M, Shimizu W, Fukuzawa M, et al. Efficacy and Safety of Edoxaban 15 mg According to Renal Function in Very Elderly Patients With Atrial Fibrillation: A Subanalysis of the ELDERCARE-AF Trial. Circulation. 2022; 145: 718-720. https://doi.org/10.1161/CIRCULATIONAHA.121.057190.

[3] Shimizu W, Yamashita T, Akao M, Atarashi H, Ikeda T, Koretsune Y, et al. Renal Function and Clinical Outcomes Among Elderly Patients With Nonvalvular Atrial Fibrillation From ANAFIE. JACC Asia. 2023; 3: 475-487. https://doi.org/10.1016/j.jacasi.2023.02.002.

[4] Yamashita Y, Morimoto T, Muraoka N, Oyakawa T, Umetsu M, Akamatsu D, et al. Edoxaban for 12 Months Versus 3 Months in Patients With Cancer With Isolated Distal Deep Vein Thrombosis (ONCO DVT Study): An Open-Label, Multicenter, Randomized Clinical Trial. Circulation. 2023; 148: 1665-1676. https://doi.org/10.1161/circulationaha.123.066360.
